# Psychometric Properties of Child (0–5 Years) Outcome Measures as used in Randomized Controlled Trials of Parent Programs: A Systematic Review

**DOI:** 10.1007/s10567-019-00277-1

**Published:** 2019-02-26

**Authors:** Nicole Gridley, Sarah Blower, Abby Dunn, Tracey Bywater, Maria Bryant

**Affiliations:** 1 Department of Health Sciences, University of York, York, YO10 5DD UK; 2 Clinical Trials Research Unit, Leeds Institute of Clinical Trials Research, University of Leeds, Leeds, UK; 3 Present Address: Carnegie School of Education, Leeds Beckett University, Leeds, UK

**Keywords:** Systematic review, Outcome measures, Social emotional development, Psychometric properties, COSMIN

## Abstract

This systematic review is one of the three which sought to identify measures commonly implemented in parenting program research, and to assess the level of psychometric evidence available for their use with this age group. This review focuses specifically on measures of child social–emotional and behavioral outcomes. Two separate searches of the same databases were conducted; firstly to identify eligible instruments, and secondly to identify studies reporting on the psychometric properties of the identified measures. Five commercial platforms hosting 19 electronic databases were searched from their inception to conducted search dates. Twenty-four measures were identified from Search 1: a systematic search of randomized controlled trial evaluations of parenting programs. For Search 2, inclusion/exclusion criteria were applied to 21,329 articles that described the development and/or validation of the 24 measures identified in Search 1. Thirty articles met the inclusion criteria. resulting in 11 parent report questionnaires and three developmental assessment measures for review. Data were extracted and synthesized to describe the methodological quality of each article using the COSMIN checklist alongside the overall quality rating of the psychometric property reported for each measure. Measure reliability was categorized into four domains (internal consistency, test–re-test, inter-rater, and intra-rater). Measure validity was categorized into four domains (content, structural, convergent/divergent, and discriminant). Results indicated that supporting evidence for included measures is weak. Further work is required to improve the evidence base for those measures designed to assess children’s social–emotional and behavioral development in this age group. PROSPERO Registration number: CRD42016039600.

**Electronic supplementary material:**

The online version of this article (10.1007/s10567-019-00277-1) contains supplementary material, which is available to authorized users.

Social–emotional and behavioral problems in infancy are common (Skovgaard et al. 2007) and are predictive of poor outcomes in later childhood (Skovgaard et al. 2008). Common risk factors for children’s poor social–emotional and behavioral development include impaired dyadic relationships, parental mental health issues, and inconsistent or inappropriate parenting behavior (Shonkoff and Phillips 2000). In the UK, and internationally, early intervention and prevention of poor social–emotional and behavioral development via parenting programs has become a dominant theme in public health initiatives for supporting children in the first 5 years of life (Allen 2011). The key objective is to improve children’s social–emotional, behavior, and cognitive development by targeting the parent as the active agent of change (Barlow et al. 2016; Furlong et al. 2012). Research indicates that group-based parenting programs can be effective and cost effective for children under five (Barlow et al. 2016; Furlong et al. 2012; O’Neil et al. 2013). For example, intervening early in a child’s life to prevent problems from escalating is estimated to incur cost savings of approximately £70,000 per individual by the time they reach 30 years old (Scott et al. 2001).

Early identification is vital to the developing child, and measures used to screen and monitor issues must be robust in reliability and validity to ensure that the support offered is appropriate to the individual’s needs. Despite this, researchers and practitioners struggle to decide which measures to adopt to monitor change as a consequence of an intervention. Often decisions are based on familiarity, or accessibility. In addition, in some situations, funding bodies stipulate when a trial should be powered on a specific parent or child outcome. As a result, the literature is awash with inconsistency, limiting generalizability between different studies, and applicability to practice where measures need to be inexpensive, and easy to implement and interpret (McCrae and Brown 2017).

Whilst there is existing guidance for selecting outcome measures to assess school age child health, there is limited guidance for younger children (i.e., 0–5 years) as well as a lack of agreement as to which measure should be accepted as the single standard (Wigglesworth et al. 2017). Traditionally standardized developmental tests (e.g., Bayley’s Scales of Infant Development; Bayley 1993, 2006), based on observation, have been considered the gold standard for establishing child outcomes. This is because they are objective, valid, and are generally considered to provide a reliable assessment of an infant’s development in comparison to norms and standardized scores (Johnson and Marlow 2006). However, several non-systematic reviews of developmental tests for this age group have indicated that often the norms applied are outdated and do not reflect the general population from which the child is drawn (Johnson and Marlow 2006). Moreover, such measures often have a distinct lack of evidence of predictive validity, and evidence to support their test–re-test reliability, concurrent, content, and construct validity is generally limited or poor (Bradley-Johnson 2001; Johnson and Marlow 2006).

Parent-reported measures of children’s outcomes are considered a less expensive, and more efficient, alternative to observational measures. Unlike standardized developmental tests, whose purpose is to diagnose, parental report is often considered useful as a screening method to identify children who may need further assessment before a diagnosis is made. Several systematic and non-systematic reviews of measures for children’s mental health and social and emotional development have indicated that very few parent report questionnaires of social–emotional and behavioral development are actually available for use with children under five (Deighton et al. 2014; Gilliam et al. 2004; Halle and Darling-Churchill 2016; Humphrey et al. 2011; McCrae and Brown 2017; Pontoppidan et al. 2017; Szaniecki and Barnes 2016). Moreover, there is a large gap for measures developed for use with children under 2 years, possibly due to minimal testing of its reliability and validity with this age group (Gilliam et al. 2004; McCrae and Brown 2017; Pontoppidan et al. 2017). Research suggests that the use of measures with younger children (birth to 3 years) that were designed for older children may not be sufficiently sensitive to rapid developmental shifts associated with this age group, and the presentation of problems in younger children may not imitate those seen in older children (Whitcomb 2012).

Although researchers claim that many measures for birth to 3 years are psychometrically robust, a series of systematic reviews in this area have identified that gaps exist for their predictive validity and test–re-test reliability (Halle and Darling-Churchill 2016; Humphrey et al. 2011; McCrae and Brown 2017). Moreover, Pontoppidan et al. (2017) warns that the majority of psychometric evidence has been extracted from technical reports written by the developers, and that independent testing is required to establish an accurate evidence base. Conflicting conclusions reached by different researchers regarding a measure’s psychometric standing indicate that the process of synthesizing evidence from multiple studies/reviews of measurement properties should be supported by a standardized method using predefined guidelines (Lotzin et al. 2015).

The current review had two aims and comprised two separate database searches. Aim 1 was to identify the most commonly reported child outcome measures used in randomized controlled trial (RCT) evaluations of parenting programs delivered antenatally and/or for parents of children up to and including 5 years. Specifically, we were interested in measures that provided an assessment of the child’s behavior, social and emotional development, and cognitive outcomes. Aim 2 was to identify and synthesize the current evidence base for each of the included measures psychometric properties via a second systematic search of the scientific literature. The rationale for focusing specifically on commonly used measures within RCTs of parenting programs was twofold. Firstly, we wished to identify the breadth of child outcome measures being commonly adopted for evaluation purposes, and secondly, we sought to recommend a small battery of reliable and valid outcome measures that could be used by both researchers and practitioners seeking to evaluate change. Throughout the remainder of this review, evidence for each of the included measures psychometric standing will be conceptually organized according to their reliability and validity using the terms and definitions applied by the COSMIN checklist (de Vet et al. 2015; Terwee et al. 2007).

## Method

This review included two distinct search stages. Search 1 identified RCTs of parenting programs for parents of children from the antenatal period up to the child’s fifth birthday published in the scientific literature. From these studies, measures of child outcomes which had been used to evaluate the intervention were extracted. Measures which were identified as having been used in three of more of the retrieved RCTs were then included in search two. The purpose of Search 2 was then to identify papers describing the development and subsequent validation of these measures via an additional database search.

### Domain Map

In preparation for the systematic review, two researchers (SB & TB) undertook a domain-mapping exercise as recommended by Vaughn et al. (2013). The intention was to enable classification of identified outcome measures by population of interest. Outcome domains were mapped under three categories; parent, child, and dyadic. The current review focuses solely on the child domain, resulting in parent-reported questionnaires and practitioner-administered assessments for review. The findings for measures in the parent and the dyadic domains are described in two companion systematic reviews (Blower et al. 2019; Gridley et al. 2019).

### Search 1: Identifying Tools used in Parenting Program Research

#### Eligibility Criteria for Evaluation Studies

Search 1 focused solely on identifying high-quality parent program evaluations, i.e., RCTs; consequently, the literature searches were restricted only to peer-reviewed items. Included studies (1) presented primary research relating to the evaluation of a parenting program using an RCT design. Studies reported a randomly allocated treatment and comparison group (which was any comparator, e.g., control, waiting list, other treatments); (2) included samples that included expectant parents, mothers and/or fathers, or other types of primary carer, of children up to and including the age of 5 years (where the evaluation spanned a wider age range at least 80% of the participants had to meet this criterion); (3) described a parenting program that was structured, manualized, delivered by trained facilitator, and designed to improve some aspect of child social and emotional wellbeing or behavior; (4) reported on at least one relevant parent–child outcome which had been developed and validated independently of the RCT; (5) were published in the English language within the period 1995–2015. Papers were excluded if they met the inclusion criteria but (1) there was insufficient information to determine eligibility (where a scan of full text could not provide missing information), and (2) the manuscript was not available to download in full-text format from host Universities library, Endnote, Paperpile, or Google Scholar.

#### Search Strategy for Obtaining Evaluation Studies

A total of five commercial platforms hosting 19 scientific databases were searched in November 2015, with only studies published after January 1995 included because of increasing prevalence of RCTs. Databases were searched in English. An example of the search strategy used for retrieving relevant papers from each of the 19 databases is as follows:

parent* training* OR parent* program* OR parent* education OR parent* intervention* AND toddler OR infant OR pre*school OR bab*y OR child* *OR pregnancy OR antenatal* AND *experimental OR randomi?ed controlled trial*.

See online resource Fig. 1 for a flowchart depicting article retrievals. The databases that were searched were Arts and Humanities Citation Index, ASSIA, British Nursing Index, CINAHL plus, Cochrane Library, Conference Proceedings Index, DARE, Econlit, EMBASE, ERIC, HTA, Maternity and Infant care Database [MIDIRS], MEDLINE Journal articles, NHS EED, Psycarticles, PsycInfo, Social Policy and Practice database [SOPP], Social Science Citation Index expanded, and Social Sciences Citation Index.

#### Article Selection and Data Extraction

All retrieved articles were downloaded into an Endnote database and duplicates removed. Three reviewers (SB, NG, and ZH) independently performed a title and abstract screen of the remaining articles before performing a full-text screen applying the inclusion and exclusion criteria outlined above. Prior to data extraction, inter-rater reliability checks were performed on a 20% random selection of all identified and included articles, and a 20% random selection of all excluded articles by two of the three reviewers. There were no recorded disagreements between reviewers.

Three reviewers (SB, NG, and KT) independently extracted data from the remaining articles using a google form to enable consistency. Data that were extracted were study authors, study design (i.e., parallel RCT or cluster), parenting program name and type (i.e., group or one to one), country of study, sample size and characteristics (i.e., age, gender, primary caregiver, ethnicity), the reported measures, and their defined constructs according to our initial domain-mapping exercise, i.e., attachment, bonding, maternal sensitivity, parent–child interaction.

The data were then synthesized by two reviewers (SB and NG). This process sought to identify each individual measure and the number of times it occurred as an outcome in the included RCTs. The measures were then grouped within the domains, i.e., parent, child, dyadic by their format (i.e., questionnaires, developmental tests or observational tools). As the objective of Search 1 was to identify the most commonly reported measures used in RCT evaluation, it was important that measures included in Search 2 were widely used in the evaluation of parenting program research. To avoid bias that may occur by applying strict criteria, the optimal threshold of appearances was explored. Across all three domains (parent, child, and dyadic outcomes) inclusion in at least three or more independent trials proved to be the optimum cut-off, and subsequently this threshold was applied to identify the most relevant measures of interest.

### Search 2: Identifying the Development and Validation Studies of Eligible Measures

#### Eligibility Criteria

Inclusion criteria were papers which (1) described the development or evaluation of a questionnaire or developmental test identified in Search 1; (2) reported on a sample of expectant parents, mothers and/or fathers, and other types of primary carer of children up to and including the age of five (where the study population spanned a wider age range at least 80% of the participants had to meet this criterion); (3) was published in the English language; (4) was published as a full-text article; and (5) related to the most recent/short version of the measure. The exclusion criterion was that the population were a clinical subpopulation unrelated to the outcome (i.e., a group of children diagnosed with autism).

#### Search Strategy

Databases were the same as for Search 1, with the exception of Centre for Reviews and Dissemination (DARE, HTA, NHS EED) and the Cochrane Library, which were not searched. Research indicates that it can be difficult to identify articles reporting the development or evaluation of measures due to inconsistencies in the indexing and keywords used by different databases (Bryant et al. 2014). Subsequently, we drew upon a complex key search term syntax developed by Terwee, Jansma, Riphagan, and de Vet (2009) and implemented by Bryant et al. (2014) and McConachie et al. (2015). See online resource Table 1 for an example of the search strategy. Retrieved articles were then downloaded into an Endnote database. Each article was subject to a title and abstract screen. Articles meeting the initial inclusion/exclusion criteria were then subject to a full-text screen to assess eligibility for data extraction. Inter-rater reliability checks were performed on a 20% random selection of all identified and included articles retained for each tool included in the review, and a random 20% selection of all articles excluded at the full-text screen stage. Approximately, 1% of all papers resulted in a disagreement between researchers. Disagreements were resolved via consultation with a third reviewer who had not been involved in the initial screening or reliability check.

#### Data Extraction

Search 2 data were extracted and entered onto pre-determined data extraction forms using Qualtrics software. A systematic approach was taken to capture both the quality and evaluation of findings reported in eligible articles according to the structure of two sources; (1) the COSMIN (Terwee et al. 2011a) checklist, and (2) the Terwee, de Vet et al. (2011b) quality criteria for measurement properties checklist (see http://www.cosmin.nl/ for further information).

To ensure that each of the included studies met the standards for good methodological quality, and that the risk of bias was minimal, the COSMIN was used as a measure of the articles methodological quality. The COSMIN was developed via a Delphi study in response to the need for a standardized method to assess measurement studies and consistent application of psychometric definitions. Consequently, the COSMIN was selected for the purposes of the current review over other guidelines due to its advantages of standardizing cross-cultural comparisons, and its facilitation of comparisons between different measurement studies (Paiva et al. 2018). The quality of a study’s methodology is assessed according to 10-psychometric domains of interest: (1) Internal consistency (11 items), (2) Reliability (14 items), (3) Measurement error (11 items), (4) Content validity (5 items), (5) Structural validity (7 items), (6) Hypothesis testing (10 items), (7) Cross-cultural validity (15 items), (8) Criterion validity (7 items), (9) Responsiveness (18 items), and (10) Interpretability^1^ (7 items). Items across all 10-psychometric domains consider both the design (missing items and sample size) and statistical reporting (specific analysis performed) of the study using a four-point scale (i.e., poor, fair, good, or excellent). The 10 COSMIN psychometric domains are further described in Online resource Table 2. Applying the COSMIN taxonomy and definitions (de Vet et al. 2015; Terwee et al. 2007) three reviewers (SB, NG, and AD), qualified to PhD level, independently extracted data from eligible articles. Reviewers only extracted data relating to the specific psychometric domains reported in each study, that is, no study was penalized for not reporting on all 10-psychometric domains. Each reported psychometric property was then provided an overall rating for its methodological quality based on COSMIN criteria of taking the lowest rating of any item within a domain, i.e., worse score counts (Terwee et al. 2011a). Prior to data synthesis, inter-rater reliability checks were performed on 100% of the overall quality ratings. Two reviewers resolved disagreement through consensus. If no agreement could be reached, the third reviewer was asked to make a final decision.

Following completion of the assessment of methodological quality using the COSMIN, the quality of the psychometric evidence provided for each domain reported within each individual study was assessed using the Terwee et al. (2011b) checklist. This checklist mirrors the 10-psychometric domains captured by the COSMIN with findings across each domain rated on a three-point scale (positive, indeterminate, or negative). To ensure the checklist met the needs of the review, some modifications were made so that definitions were transparent and easily applied across all of the included studies (see online resource Table 2). To make certain that we did not undermine the integrity of the results by modifying a standardized measure, the final criteria included a combination of the original (2007) definitions (where the criteria have not been recently amended), more recently updated guidelines (where the 2007 definition has been recently changed), and additional criteria implemented by recent users of the checklist (where definitions were previously obsolete).

#### Data Synthesis

To provide an overall evaluation of each measure’s reported level of evidence across the 10-psychometric domains, three reviewers (NG, SB, and AD) pooled the methodological quality ratings (i.e., poor, fair, good, or excellent) from the COSMIN with the ratings applied for their reported psychometric evidence (i.e., positive (+), indefinite (?), or negative (-) ratings) using the Terwee checklist. To ensure that no measure was unfairly disadvantaged during the data synthesis stage, the following rules were applied to account for differences in the number of studies providing supporting evidence for each of the 10-psychometric domains:

**Strong Level of Evidence (+++ or −−−)**: This rating was applied when the evidence for the target psychometric property of a measure was supported by consistently positive or negative findings in multiple studies (two or more) rated good in methodological quality, or in one study of excellent methodology quality.

**Moderate Level of Evidence (++ or −−)**: This rating was applied when the evidence for the target psychometric property of a measure was supported by consistently positive or negative findings in multiple studies (two or more) rated fair in methodological quality, or in one study of good methodological quality.

**Limited Level of Evidence (+ or −)**: This rating was applied when the evidence for the target psychometric property of a measure was supported by positive or negative findings from one study rated fair in methodological quality.

**Conflicting Level of Evidence (+/)**: This rating was applied when the evidence for the target psychometric property of a measure was supported by studies with conflicting findings.

**Unknown (?)**: This rating was applied when the evidence for the target psychometric property of a measure was supported only by studies of poor methodological quality, or the criteria were not met for a positive or negative rating in the majority of reviewed studies.

## Results

Search 1 yielded 16,761 articles, with 279 articles progressing to the data extraction stage (see online resource Fig. 1). The 279 articles comprised peer-reviewed and published RCTs describing the evaluation of 113 parenting programs delivered within clinics or communities as one-to-one or group-based programs. Sample characteristics varied across individual studies in terms of size (i.e., range *N* = 24 to 5563), target caregiver (e.g., mothers only, or mothers and fathers), ethnicity and country of study, indicating that this pool provided an adequate representation of the available literature. A total of 480 measures were reported across the 279 studies including questionnaires (*N* = 268), developmental tests (*N* = 55), observational tools (*N* = 106), and other formats (*N* = 51) such as clinical interview schedules. Assessment of the varying frequencies of use/occurrence of measures across independent RCTs (≥ 1, ≥ 2, ≥ 3, ≥ 4) was conducted to determine the optimal criteria that best represented the term ‘commonly used.’ Application of these thresholds across all three domains (parent, child, and dyadic) indicated that ≥1 and ≥2, yielded too many measures for the review to be manageable and meaningful, whilst the difference between the ≥3 and ≥4 criteria was minimal. Subsequently, three or more appearances were deemed appropriate for all domains and these criteria were applied leaving 17 parent report questionnaires and seven developmental tests eligible for progression to Search 2.

Initial database searches for Search 2 returned 21,329 papers (see online resource Fig. 2). Following a title and abstract screen, 5,669 duplicates were removed and a further 15,117 papers were found to be ineligible. Of the remaining 543 articles sent for full-text screen, 513 were excluded leaving 30 articles representing 11 questionnaires and three developmental tests for data extraction. Characteristics of the 14 measures are described in Table 1; those of each study are described in Table 2. The final synthesized evidence for each measure’s psychometric properties is provided in online resource Table 3. A summary of the psychometric evidence for the measures identified is described below in the following order; (1) parent-reported measures of child behavior, (2) social–emotional development, (3) language development, and finally, (4) Practitioner-administered developmental tests.

**Table 1:** Summary characteristics of all measures included in the systematic review

Tool Author(s) (Date)	Age	Subscales	Items	Response options	Scores		Completion Time (min)	Administration format	Availability	Costs^a^
Parent-reported child behavior measures										
CBCL 1.5-5 Achenbach and Rescorla, (2000)	1.5-5 years	2	99	3	0–198		10	Paper	Licensed by publisher: http://store.aseba.org/MANUAL-FOR-ASEBA-PRESCHOOL-FORMS-PROFILE/productinfo/605/	Manual: $45 Pack of 50 forms: $30
CBRS Bronson et al. (1990)	3–5 years	2	32	5	32–160		5–10	Paper	Items available in original research articles	Not known
ECBI Eyberg and Ross (1978) Eyberg and Pincus (1999)	2–16 years	2	36	7	36–252		5	Paper/Electronic systems for administration/scoring also available	Available from measure publisher http://www.parinc.com	Manual: $65 Pad of 25 forms: $49
IBQ-R Gartstein and Rothbart (2003)	3–12 months	14	91	7	0–637		10	Paper	Available from developer website https://research.bowdoin.edu/rothbart-temperament-questionnaires/	Free (must register to download form)
SDQ 2–4 Goodman (1997)	2–4 years	5	25	3	0–40		5	Paper	In public domain http://www.sdqinfo.com/	Free
SDQ 4–17 Goodman (1997)	4–17 years	5	25	3	0–40		5	Paper	In public domain http://www.sdqinfo.com/	Free
BIQ Bishop, Spence, and McDonald (2003)	3–5 years	6	30	7	30–210		10	Paper	In public domain http://www.scaswebsite.com/index.php?p=1_56	Free
BITSEA Briggs-Gowan and Carter (2002; 2006)	12–36 months	2	42	3	0–126		7–10	Paper	License required from publisher http://www.pearsonclinical.co.uk	Kit $170.71
PAS-R Spence et al. (2001)	3–6 years	5	30	5	0–120		5–10	Paper	Available to download from developer website https://www.scaswebsite.com	Free
MCDI Level 1 and 2 Fenson et al (1993)	8–30 months	2	189	Checklist/Yes/No	0–189		15–20	Paper	From developer http://www.brookespublishing.com	$99.99
MCDI Level 3 Fenson et al (2007)	30–37 months	3	124	checklist/yes/no	0-124		15–20	Paper	From developer http://www.brookespublishing.com	Manual $59.95 Downloadable forms $51
Practitioner-administered developmental tests										
BSID-III Bayley (2006)	1–42 months	5	N/K	Yes/no	N/K	35–60		Paper	From publisher http://www.pearsonclinical.com	Kit $1,248.00
MSEL Mullen (1995)	0–68 months	5	N/K	Yes/No	N/K	35–60		Paper	From publisher: http://www.pearsonclinical.com	Kit $956.80
NRDLS Edwards et al. (2011)	3–7 years	2	N/K	Yes/no	N/K	35–60		Paper	From publisher: http://reynell.gl-assessment.co.uk/	Kit $808.33

*N/K* not known

^a^Costs obtained in January 2018

**Table 2:** Sample characteristics of included studies grouped by outcome domain specified in the review

Instrument	Author (Date) Country	*N*	Parent Age in years (*SD*)	Child Age (*SD*)	% Female (Parent)	% Female (Child)	Predominant Ethnicity (%)	Study setting	Recruitment methods
Parent-reported child behavior									
CBCL 1.5-5	Cai et al. (2004) USA	614	28.3 (8.76)	3.6 years (0.30)	88	49	African American (88)	General population/Community (Head Start)	Convenience
	Tan et al. (2007) USA	757	N/K	3.24 years (1.26)	N/K	100	Parents, Caucasian (95); Children, Chinese (100)	General Population of Chinese adopted children	Selected from larger sample of available data
CBRS	Mui Lim et al. (2010a) Singapore	117	N/A	5 years (10 months)	N/A	48	Chinese (76.1)	General population/Community	Convenience
	Mui Lim et al. (2010b) Singapore	117	N/A	5 years (10 months)	N/K	48	Chinese (76.1)	General population/Community	Convenience
	Mui Lim et al. (2011) Singapore	117	N/A	5 years (10 months)	N/A	48	Chinese (76.1)	General population/Community	Convenience
	Schmit et al. (2014)USA	247	N/K	31.01 months (4.30)	N/K	50	Caucasian (60)	General population/Community Longitudinal study of Head Start	Convenience
ECBI	Butler (2011) USA	47–397	N/K	Range 3–6 years	100	Study A = 47; Study B = NK	African American (70)	General population/Community	Convenience - drawn from a larger study
	Funderburk et al. (2003) USA	88	N/K	53 months (12.3)	100	49	Caucasian (100)	General population/Community	Convenience/Random
	Gross et al. (2004) USA	241	N/K	Range 2–4 years	88	43	African American (58.9)	General population/Community - Preschool	Convenience
	Gross et al. (2007) USA	682	30.8 (7.4)	3 years, Range 2–4 years	83	51.5	Latino (46.8)	General population/Community	Convenience
	Rich and Eyberg (2001) USA	198	N/K	4.38 (1.01)	N/K	20	Caucasian (78)	General population/Community/Clinic	Sample matching
	Weis et al. (2005) USA	Study 1 489; Study 2 115	Study 1 = 31.06 (6.51) / Study 2 N/K	Study 1 = Range 2–6 years / Study 2 = Range 4–6 years	100	Study 1 = 47; Study 2 = 33	Caucasian (86)	General population/Community/Clinic/Referred	Convenience
IBQ-R	Gartstein and Rothbart (2003) USA	360	N/K	Range 3–12 months	90	50	Caucasian (NK)	General population/ Community	Convenience
	Giesbrecgt and Dewey (2014) Canada	458	31.2 (4.1)	Time 1 = 2.7 months; Time 2 M = 6.2 months	100	44	Caucasian (85.7)	General population/Community	Convenience
SDQ 2–4	Croft et al. (2015) England	16,659	N/K	3.15 years	NK	52	N/K	General population/Community - drawn from large cohort study	Convenience
	D’Souza et al (2016) New Zealand	6822	31	2 years	100	48	Caucasian (67.4)	General population/Community	Convenience
SDQ 4–17	Chiorri et al. (2016) England	695	N/K	51 months	50	N/K	N/K	General population/Community - drawn from large cohort study	Convenience
	Dave et al. (2008) England	NK	Fathers 39.8 (5.5); Mothers 37.62 (4.69)	4–6 years	50	47	Caucasian (85%)	General population/Community	Convenience
	Kremer et al. (2015) Australia	53,372	N/K	Range 4–6 years	85	49	Australian (90.2)	General population/Community	Convenience
Parent-reported child social and emotional development									
BIQ	Kim et al (2011) USA	495	N/K	3.5 years (0.3 months)	50	46	Caucasian (87)	General population/Community	Convenience
BITSEA	Briggs-Gowan et al. (2004) USA	1280	27.5 (5.4)	23.8 months (6.8)	96	51	Non-Hispanic white (66.3)	General population/Community	Random from list of birth records of babies in specific area
	Briggs-Gowan and Carter (2007) USA	192	Mothers 32.9 (8.7); Fathers 35.5 (7.4)	26.1 months (6.1)	85	25	Caucasian (56.3)	Referred/Community	Convenience
	Briggs-Gowan et al. (2014) USA	260	N/K	34.9 months (6.8)	86	28	Caucasian (45.5)	Referred/Community	Convenience
PAS-R	Edwards et al. (2010) Australia	764	Mothers 35.81 (4.47); Fathers 38.09 (5.19)	47.39 months (6.37)	93	50	Caucasian (85.5)	General population/Community	Convenience
Parent-reported child language development									
MCDI levels i and ii	Fenson et al. (2000) USA	1394	N/K	Range 8–34 months	N/K	N/K	Caucasian Infant (88.7); Toddler (92.5)	General population/Community	Convenience
MCDI level iii	Skarakis-Doyle et al. (2009) USA	58	N/K	TDL = 369.51 (3.38); LI = 39 (6.14)	N/K	TDL = 61; LI = 22	Caucasian (NK)	General population/Community/Referrals	Convenience
Practitioner-Administered Developmental Test									
BSID-III	Connolly et al. (2012) USA	48	N/K	Range 29 days to 25 months	N/K	33.30%	Caucasian (66.7%)	Clinic	Convenience
	Moore et al. (2012) UK	185	N/K	Range 29 to 41 months	N/K	51%	N/K	General population/Community	Convenience
MSEL	Farmer et al. (2016) USA	118	N/K	63.67 months	N/K	28%	Caucasian 79%	Primary care	Convenience
NRDLS	Letts et al. (2014) UK	301 and 1266	N/K	Between 1:06 and 7:06 for development and 2 and 7 for validation	N/K	Approx 50%	N/K	General population/Community	Convenience

*N*/*A* Not applicable, *N*/*K* Not known, *TDL* typically developing language, *LI* Language impairment, *CBCL* Child Behavior Checklist, *CBRS* Child Behavior Rating Scale, *ECBI* Eyberg Child Behavior Inventory, *IBQ-R* Infant Behavior Questionnaire-Revised, *SDQ* Strengths and Difficulties Questionnaire, *BIQ* Behavioral Inhibition Questionnaire, *BITSEA* Brief Infant Toddler Social and Emotional Assessment, *PAS-R* Preschool Anxiety Scale Revised, *MCDI* MacArthur Bates Communication Development Inventories, *BSID* Bayley Scales of Infant Development, *MSEL* Mullen Scales of Early Learning, *NRSLD* New Reynell Developmental Scales of Language

### Parent-Reported Measures of Child Behavior

Six measures of parent-reported measures of child behavior were identified and reviewed, namely, the Child Behavior Checklist (CBCL; Achenbach and Rescorla 2000), the Child Behavior Rating Scale (CBRS; Bronson et al. 1990), the Eyberg Child Behavior Inventory (ECBI; Eyberg and Ross 1978; Eyberg and Pincus 1999), the Infant Behavior Questionnaire—Revised (IBQ-R; Gartstein and Rothbart 2003), and the Strengths and Difficulties Questionnaire 2–4, and 3-16-year versions (SDQ; Goodman 1997).

Internal consistency assessments were reported for all six measures. Overall, the CBCL (Tan et al. 2007), ECBI (Butler 2011; Gross et al. 2004, 2007; Weis et al. 2005), SDQ 2–4 (Croft et al. 2015; D’Souza et al. 2016), and SDQ 3–16 years (Dave et al. 2008; Kremer et al. 2015) provided the strongest evidence. One study rated fair in methodological quality reported on the CBRS (Schmitt et al. 2014). Whilst using only the 10 items that comprised the behavioral self-regulation factor, alphas exceeded the Terwee criteria of > 0.70 yielding a positive value with limited evidence for its psychometric property. Finally, whilst two studies reporting on the IBQ-R met Terwee criteria, rated poor of methodological quality for this aspect of the study, the overall psychometric evidence was rated as unknown (Gartstein and Rothbert 2003; Giesbrecht et al. 2014).

Test–re-test reliability was only reported for the ECBI in one study of fair methodological quality (Funderburk et al. 2003). Results failed to meet Terwee criteria (Pearson’s *r* > .80) over a ten-month period yielding a negative rating with limited evidence for this psychometric property. Finally, inter-rater reliability estimates were reported for the IBQ-R and the SDQ 3–16 years by comparing primary and secondary caregiver reports (Chiorri et al. 2016; Dave et al. 2008; Gartstein and Rothbert 2003). Over 50% of the analyses for the IBQ-R failed to reach the Terwee threshold (ICC/weighted Kappa > 0.70 OR Pearson’s *r* > .80), and rated poor in methodological quality rendered an unknown rating for this psychometric property. Neither study that reported data for the SDQ 3–16 years met Terwee criteria, resulting in a moderate level of evidence of inter-rater reliability with negative findings for this measure.

Content validity was only reported for the IBQ-R in one study rated as good in methodological quality (Gartstein and Rothbart 2003). A multi-phase scale construction method was used which included the generation of operational definitions followed by evaluation of item content via a group of experts. Item analysis was then conducted on the 16 scales by age groups reducing the number of items by almost half. Item-total correlations reached 0.30, yielding a moderate level of positive evidence for this psychometric property.

Structural validity was reported for all child behavior measures with the strongest evidence for the CBRS (Mui Lim et al. 2010a, b). By contrast, the factor structure of the CBCL (reported in Tan et al. 2007), ECBI (Butler 2011; Gross et al. 2007; Weis et al. 2005), the SDQ 2–4 (Croft et al. 2015; D’Souza et al. 2016), and the SDQ 3–16 (Chiorri et al. 2016) performed poorly against Terwee criteria (factors should explain at least 50% of the variance OR CFI or TLI or comparable measure > 0.95 AND (RMSEA < 0.06 OR SRMR < .08)) rendering moderate to strong levels of evidence for negative findings. Evidence to support the factor structure of the IBQ-R was rated unknown as the overall variance explained by the model reported in Gartstein and Rothbert (2003) was not presented.

Convergent/divergent validity was only reported for the CBCL, CBRS, and the ECBI. For the CBCL, a study comparing parent report with the teacher report version failed to reach the Terwee threshold (*r* > .50) yielding a limited level of evidence with negative findings for this psychometric property (Cai et al. 2004). Similarly, a study reporting comparisons between the CBRS and the Evaluation of Social Interaction measure (ESI: Fisher and Griswold 2009) also failed to meet Terwee criteria yielding a limited level of evidence with negative findings (Mui Lim et al. 2010a). Conversely, assessments between the ECBI and CBCL in two studies (Butler 2011; Gross et al. 2007) and the Preschool Behavior Questionnaire (PBQ-P: Behar and Stringfield 1974) in one study (Funderburk et al. 2003) yielded moderate levels of evidence with positive findings for convergent validity.

Criterion validity was only reported for the ECBI. In one study of good methodological quality (Rich and Eyberg 2001) and one of fair (Weis et al. 2005), results indicated that the ECBI has good levels of sensitivity and specificity with both the DSM-III revised structured interview criteria for diagnosis of disruptive behavior disorders, and the Disruptive Behavior Disorder Rating Scales (DBDRS: Barkley 1997), yielding a moderate level of evidence with positive findings for this psychometric property.

### Summary of Parent-Reported Measures of Child Behavior

None of the behavior measures performed consistently well across multiple measurement properties. The evidence reviewed suggests that the strongest support can be found for the CBCL, SDQ 2–4, and 3–16 years in terms of internal consistency; the CBRS is stronger in structural validity; and the ECBI has greater evidence for its convergent/divergent and criterion validity.

### Parent-Reported Measures of Social and Emotional Development

Three measures of child social and emotional development were identified and reviewed: Behavioral Inhibition Questionnaire (BIQ; Bishop et al. 2003), Brief Infant Toddler Social and Emotional Assessments (BITSEA; Briggs-Gowan and Carter 2002, 2006), and the Preschool Anxiety Scale Revised (PAS-R; Spence et al. 2001).

All studies for the BIQ (Kim et al. 2011), BITSEA (Briggs-Gowan et al. 2004; Briggs-Gowan and Carter 2007), and the PAS-R (Edwards et al. 2010) reported evidence for high levels of internal consistency and subsequently met criteria (alpha > 0.70) for moderate to strong levels of evidence with positive findings. Test–re-test reliability was only reported for the BITSEA. In one study rated good of methodological quality, the BITSEA demonstrated correlations over a 10- to 45-day period which met Terwee criteria (Pearson’s *r* > .80) for moderate levels of evidence with a positive rating (Briggs-Gowan et al. 2004). Inter-rater reliability was reported for all three measures. Correlations between primary and secondary caregivers on the BITSEA (Briggs-Gowan et al. 2004) and the PAS-R (Edwards et al. 2010), and parents and teachers on the BIQ (Kim et al. 2011) did not meet the Terwee threshold (ICC/weighted Kappa > 0.70 OR Pearson’s *r* > .80) meaning that all measures yielded moderate levels of negative evidence for this psychometric property.

All three measures were investigated for structural validity, with varying outcomes. Firstly, it was not possible to rate the methods and findings reported in Briggs-Gowan and Carter (2007) for the BITSEA due to a lack of reporting. Consequently, an overall evidence rating of ‘unknown’ was applied. The model reported in Kim et al. (2011) for the BIQ did not meet the Terwee threshold (CFI or TLI or comparable measure > 0.95 AND (RMSEA < 0.06 OR SRMR < .08) but rated excellent in methodological quality; this measure was awarded a strong level of evidence for negative findings for this psychometric property. Conversely, Edwards et al. (2010), rated excellent in methodological quality, reported a model for the PAS-R that did meet the Terwee criteria yielding a strong level of evidence with positive ratings for this psychometric property.

Mixed findings, relating to convergent validity, were found across the three measures. Edwards et al. (2010) reported significant correlations for both mother and father reports between the PAS-R and the SDQ Emotion problem subscale. However, the analyses failed to meet Terwee criteria (correlations with instruments measuring the same construct > 0.50 OR at least 75% of the results in accordance with the hypotheses AND correlations with related constructs are higher than with unrelated constructs) thus yielding limited levels of evidence with negative ratings for this psychometric property. Similarly, Kim et al. (2011) indicated that whilst correlations between the BIQ and related constructs on the Children’s Behavior Questionnaire (CBQ; Rothbart et al. 2001), Children’s Social Preference Scale (CSPS; Coplan et al. 2004), Preschool Age Psychiatric Assessment (PAPA: Egger et al. 1999), and Laboratory Temperament Assessment (LABTab: Goldsmith et al. 1995) were larger than with non-related constructs, less than 75% of the analyses met the Terwee criteria for a positive rating. Conversely, two studies (Briggs-Gowan et al. 2004; Briggs-Gowan and Carter 2007) reporting comparisons between the BITSEA with the CBCL 1.5–5 years indicated significant correlations for the Problems subscale and not the Competence subscale. The results were in accordance with the hypothesis and the magnitude of the correlations met Terwee criteria for moderate levels of evidence with positive ratings for its convergent/divergent validity.

Finally, criterion validity was only examined for the BITSEA in one study (Briggs-Gowan et al. 2014) against the PAPA (Egger and Angold 2004). The study was rated good in methodological quality and results met the Terwee criteria (sensitivity and specificity > 70%); however, a moderate level of evidence with a negative rating was provided as the comparator is not considered a gold standard.

### Summary of Parent-Reported Measures of Social and Emotional Development

Overall, the PAS-R appears to have the strongest evidence for internal consistency and structural validity, but the BITSEA appears to be the most robust for test–re-test reliability and convergent/divergent validity.

### Parent-Reported Measures of Child Language

Only two measures of parent-reported child language were identified for the review: the MacArthur Bates Communication Development Inventory (MCDI; Fenson et al. 1993, 2007) MCDI Level I and II (eight to 30 months), and MCDI Level III (30 to 37 months).

Internal consistency of the MCDI Levels I and II was assessed in one study rated excellent in methodological quality (Fenson et al. 2000) and the MCDI Level III in one study of good methodological quality (Skarakis-Doyle et al. 2009). Findings from both studies reached the specified criteria (alpha > 0.70) yielding moderate to strong levels of evidence with positive ratings for both measures.

Discriminant validity analysis was only assessed for the MCDI Level III in one study rated fair in methodological quality (Skarakis-Doyle et al. 2009). The findings met Terwee criteria (difference in scores on the measurement instrument for all evaluated patient subgroups is statistically significant OR > 75% of results in accordance with hypotheses) yielding limited evidence with positive ratings for this psychometric property. Criterion validity of the MCDI Levels I and II was assessed in one study rated of excellent methodological quality (Fenson et al. 2000). Results indicated a strong level of evidence with positive findings for criterion validity when comparing the short versions of the MCDI I and MCDI II against the longer versions.

### Summary of Parent-Reported Measures of Child Language

The MCDI I and II demonstrate good internal consistency and criterion validity whilst the MCDI III also demonstrates good internal consistency, as well as good discriminant validity. Subsequently, the evidence base for these measures indicates that they are both reliable and valid for use with children aged from eight to 37 months.

### Practitioner-Administered Developmental Tests

Three practitioner-administered developmental tests were identified for review: Bayley Scales of Infant Development (BSID-III; Bayley 2006), Mullen Scales of Early Learning (MSEL; Mullen 1995), and the New Reynell Developmental Scales of Language (NRSLD; Edwards et al. 2011).

Unknown ratings of evidence were applied to both the internal consistency of the NRSLD (Letts et al. 2014) and inter-rater reliability assessments of the BSID-III (Moore et al. 2012). Whilst both results met Terwee criteria, the methodological quality of the studies were poor rendering the findings inconclusive. The test–re-test reliability of the NRSLD (Letts et al. 2014) failed to meet Terwee criteria yielding a negative rating for this psychometric property.

Convergent validity assessments were provided for all three measures. Our synthesis suggests that the two studies reporting evidence for both the BSID-III (Connolly et al. 2012) and MSEL (Farmer et al. 2016) demonstrate limited to moderate evidence of convergent validity with positive findings (large correlations in expected directions) with comparable measures (such as the Differential Ability Scales II (DAS-II: Elliot 2007) and the Peabody Developmental Motor Scales (PDMS-II; Folio and Fewell 2000)). Conversely, unknown ratings were applied to the NRDLS (Letts et al. 2014). Whilst correlations with the British Picture Vocabulary Test 3rd Edition (BPVS III: Dunn and Dunn 2009) and the Test of Reception of Grammar 2nd Edition (TROG II: Bishop 2003) met Terwee thresholds, the study was rated poor in methodological quality rendering the level of evidence as unknown.

Discriminant validity analyses were conducted between a sample of typically developing and language impaired children using the NRDLS (Letts et al. 2014). The analysis met Terwee criteria; however, due to a rating of fair methodological quality, the overall evidence was deemed limited evidence with positive ratings for this psychometric property. Finally, one study of the BSID-III (Moore et al. 2012) explored its criterion validity with the Bayley 2nd edition (BSID-II; Bayley 1993). Whilst the results met the criteria (sensitivity and specificity > 70%), the BSID-II cannot be considered a gold standard. Subsequently, an unknown value for this psychometric property was provided.

### Summary of Practitioner-Administered Developmental Tests

More research is needed on all three of the development tests in order to be able to draw definitive conclusions about the performance of each instrument against key measurement properties.

## Discussion

The purpose of the current review was to identify and appraise the most commonly used child (birth up to and including 5 years) social–emotional and behavior outcome measures reported in RCT evaluations of parenting programs, in order to assess the quality and strength of their psychometric standing. The objective of this was to be able to inform the development of a small battery of recommended measures to monitor change following intervention. The review finds that despite their popularity, there is a lack of consistent evidence published by independent researchers to support the use of these measures with young children. Consequently, we were unable to propose a list of measures that could be considered for recommendation. There is a need for further assessment of the psychometric properties of child outcomes in this area to ascertain their appropriateness with this age group.

The synthesized evidence of the included measures indicates that none performed consistently well across multiple measurement properties. Evidence for the behavior measures suggests that the strongest support was found for the ECBI, SDQ, and CBRS across different psychometric domains; however, there are costs attached to the use of the ECBI which may limit its widespread use by practitioners. Conversely, the SDQ can be downloaded and used freely, whilst items from the CBRS can be obtained via published articles. The BITSEA and the PAS-R, a measure of child anxiety, appeared to have the most robust psychometric evidence for those measures representing the social–emotional domain. Usefully, the PAS-R is available in the public domain at no cost, complete with scoring instructions, whilst the BITSEA can be obtained from the publishers at a cost. The only parent-reported measure of child cognitive outcomes was the MCDI, a specific measure of child language development. Whilst lacking evidence to support all psychometric domains, those properties assessed indicated positive results. Moreover, its availability with a one-off fee makes it more feasible to researchers and practitioners for use as a language-screening measure. In terms of practitioner-administered measures of cognitive development, the findings indicate little evidence to support their psychometric standing. Consequently, further research is needed to draw definitive conclusions about the performance of each instrument against key measurement properties. This is particularly important given that the costs associated with these measures are the highest of all those included in this review.

The general lack of evidence across all psychometric domains for the included measures supports previous reviews in this area (Lotzin et al. 2015). The criteria adopted in the study to appraise both the methodological quality (COSMIN; Terwee et al. 2011a) and findings (Terwee checklist adapted from Terwee et al. 2011b) of development and validation papers are stringent and were noted to conflict with the thresholds reported by the authors of the validation studies themselves. This anecdotal finding supports conclusions from other studies that have highlighted a lack of agreement in the literature around the definitions and acceptable thresholds relating to measure reliability and validity (Lotzin et al. 2015). Both sets of standards adopted for the current review were developed in consultation with experts and agreed by consensus, thus there is a strong argument for greater investment in their use.

In line with previous research, internal consistency and structural validity were the most commonly reported psychometric properties (Lotzin et al. 2015). All parent-reported measures of child behavior and social–emotional development were supported by at least one study reporting such analysis reflecting the ease with which such assessments can be performed. Conversely, practitioner-administered developmental tests lacked sufficient evidence for most psychometric domains. It is likely that the exclusion of data published outside of peer-reviewed journals accounts for this effect with initial validation data typically presented within technical manuals or reports (Pontoppidan et al. 2017). Measurement selection should be conducted with much thought, and consideration should be given to stability over time, correlations with gold standard measures that predict longer-term trajectories for individuals, sensitivity to change, and responsiveness to intervention (Deighton et al. 2014). Where data are missing, well-informed decisions cannot be made. This is particularly concerning when researchers and practitioners wish to assess change over time following the implementation of an intervention, as without evidence to indicate a measures general level of test–re-test reliability one cannot be confident that any change observed is a direct result of the intervention, or the expected fluctuation in the measures stability over time. Consequently, further work to establish these parameters should be undertaken independently of the measure developers to ensure that measures are being tested in optimal conditions, i.e., impartially and without conflict of interest.

This review adopted independently developed and rigorous criteria to assess both the methodological quality and performance of measures. A further key strength of this review is that it provides a comprehensive assessment and synthesis of peer-reviewed, published psychometric evidence to support commonly used child outcome measures reported in RCT evaluations of parenting programs designed specifically for parents with children aged from birth to 5 years. The decision to focus on measures commonly adopted as outcomes in RCTs was to build existing consistency in the field but also because we assumed these to be the most robust measures available and most likely to be used in practice. However, the review indicates discrepancies between commonly held assumptions about the appropriateness of measures that are deemed valid and reliable because they are widely used in parent evaluations, and the current body of evidence to support their use with this age group.

Despite the rigor with which the review was conducted it is not without its limitations. The adoption of the COSMIN and Terwee checklists, even in modified form, was challenging and several issues arose around the standardization of decision making during the synthesis process. For example, the greater the number of studies assessing a psychometric property, the greater the likelihood that a conflicting evidence/indeterminate rating would be assigned. In response to this, we developed our own approach for weighting findings according to the methodological quality of studies.

Secondly, the exclusion of technical manuals may have contributed to the gaps in our knowledge for some measures, and may have skewed our conclusions regarding our ability to propose a battery of measures for both researchers and practitioners. However, technical manuals were excluded for several reasons, for example, we were unable to review all associated literature due to time constraints and we did not have funding to cover the costs associated with obtaining manuals. Whilst we acknowledge this as a limitation, we also argue that in real-world scenarios, researchers and practitioners are unlikely to be able to afford access to several technical manuals in order to be able to identify which key psychometrics render a measure more suitable for specific populations.

There is an increasing need for practitioners and researchers to evidence impact of commissioned parenting programs due to decreases in funding for child and family services both in the UK and internationally (Jerosch-Herold 2005; Roberts et al. 2014). Careful consideration needs to be given when selecting measures to assess change to ensure that they target constructs that are relevant to the program of interest, and evidence good levels of reliability and validity, whilst being time and cost appropriate for their use. The current review indicates that further research is required to establish a reasonable body of evidence to support all aspects of a measures psychometric robustness when used with the youngest children in society. The current article is important given that the findings indicate weak psychometric evidence to support some of the most popular and routinely used measures of child behavior and social and emotional development in research and practice. The current evidence base to support the use of parenting programs for parents of very young children is limited, and it is important that the measures that researchers and practitioners have available to them are robust enough to identify change following intervention where there is some.

The findings of this review suggest that very few routinely used measures have been tested and validated appropriately with this age range. Healthy development during infancy and early childhood requires competency in multiple domains. Development across those domains is inter-related but may progress at different rates (Darling-Churchill and Lippman 2016). This poses challenges for the measurement of outcomes. Across and within domains, there is a normal heterogeneity of development, making it difficult to form global judgements or ascertain typical or atypical development. This is particularly challenging for those seeking to measure outcomes from parenting programs where the age range of children covers 1–3 years (e.g., the Incredible Years Toddler program). Within domains, there are multiple potential constructs to measure, and research is still in the process of identifying those that have the strongest continuities from infancy to adulthood. Previous studies have highlighted a lack of measures that incorporate an assessment of strengths in this early age; the focus tends to be on difficulties or symptoms of developmental or other disorders (Cabrera and Tamis-LeMonda 2013; Campbell-Sills et al. 2006). Both are important. In most instances, children under 5 years of age are unable to self-report in relation to their health and development. Thus, measures typically rely on parent/caregiver report. This can be problematic in instances when parents are the recipients of the intervention being evaluated—their reports or judgements may be biased. For older children, aged 3–5 years, some measures can be rated by early childhood educators giving a different perspective. However, studies have revealed that the perspectives and ratings of both these types of rater tend not to correlate with one and other (each has a different relationship with the child, in a different context, for a different length of time and educators are more likely to make relative judgements for all the children in their care). These observations cause complications in establishing the validity and reliability of measures for this age group, particularly in terms of convergent validity and inter-rater reliability.

Consequently, we recommend that specific attention should be given to testing the responsiveness and sensitivity to change of the most promising measures identified herein. This line of research should be prioritized over and above the development of new measures, and researchers should continue to refine existing measures wherever possible. Only once this work is achieved will researchers be in a position to recommend a battery of measures appropriate for the evaluation of parenting programs. This should be regarded as an important long-term objective for researchers in the field in order to mitigate inconsistency in measure use, enhance comparability between studies and interventions, and ensure that future messages for policy-makers and practitioners are clear and transparent.

## Electronic supplementary material

Below is the link to the electronic supplementary material.

Supplementary material 1 (DOCX 85 KB)

## Footnotes

1 The items relating to interpretability are extracted solely for the purposes of study description and do not contribute to a measure’s overall quality rating.
