# Supplementary material for: Psychometric Properties of Child (0–5 Years) Outcome Measures as used in Randomized Controlled Trials of Parent Programs: A Systematic Review
Source: Clin Child Fam Psychol Rev. 2019 Feb 26;22(3):388–405. doi: 10.1007/s10567-019-00277-1 (PMC6669186; doi:10.1007/s10567-019-00277-1)
Supplement: Supplementary file 1 — Supplementary material 1 (DOCX 85 KB) [file 10567_2019_277_MOESM1_ESM.docx]

**Psychometric Properties of Child (0-5 Years) Outcome Measures as used in Randomized Controlled Trials of Parent Programs: A Systematic Review**

**Clinical Child and Family Psychology Review**

Nicole Gridley, Sarah Blower, Abby Dunn, and Tracey Bywater

Department of Health Sciences, University of York

Maria Bryant

Leeds Institute of Clinical Trials Research, University of Leeds

Correspondence concerning this article should be addressed to Sarah Blower, Department of Health Sciences, University of York, York, UK, YO10 5DD. E-mail: [sarah.blower@york.ac.uk](mailto:sarah.blower@york.ac.uk)


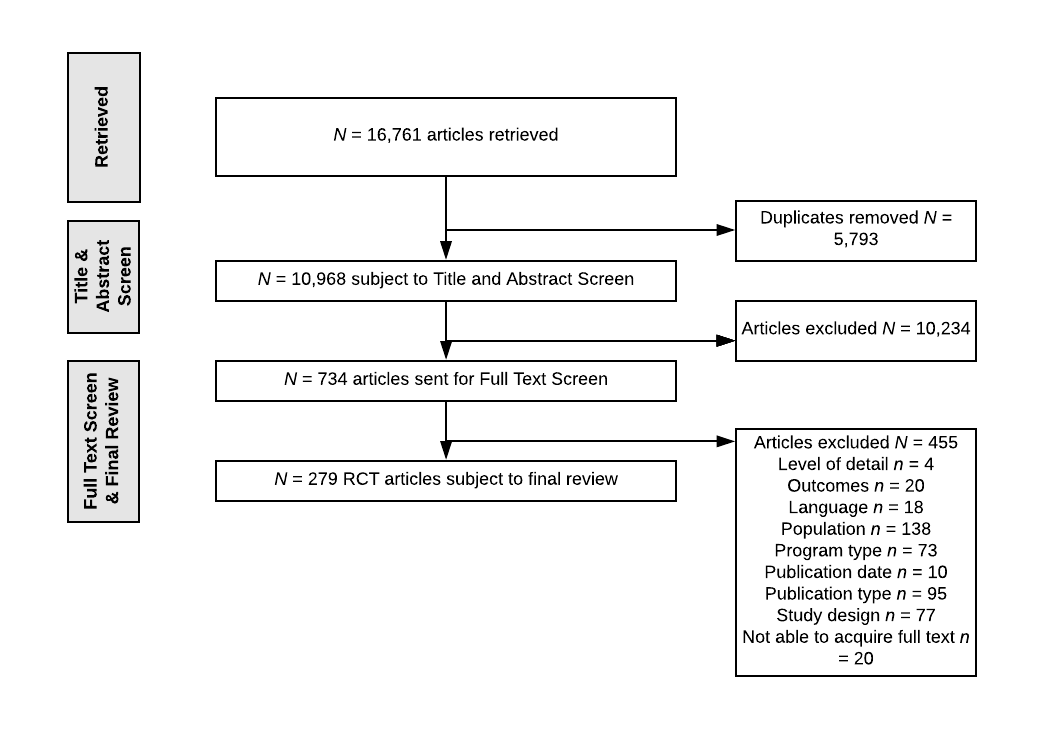


Online Resource Figure 1. *Flowchart of retrieved articles for Search 1*


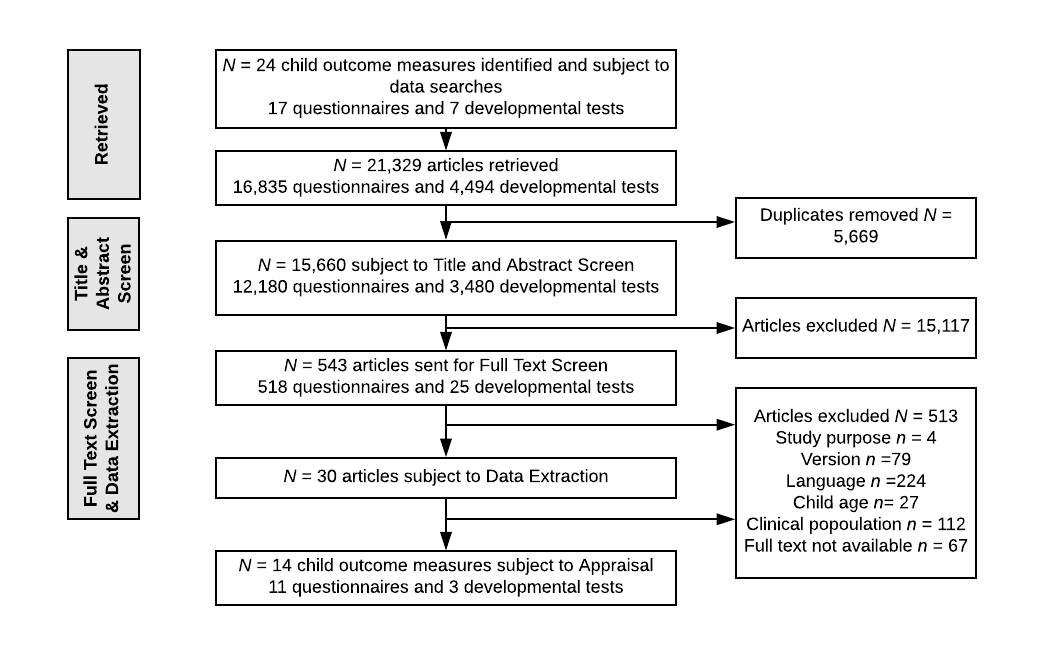


Online Resource Figure 2. *Flowchart of retrieved articles for Search 2*

*Online Resource Table 1.*

Example of search syntax used to identify eligible articles

| Search Term |
| --- |
| (Child Behaviour Checklist) AND (instrumentation.sh. OR methods.sh. OR Validation Studies.pt. OR Comparative Study.pt. OR psychometrics/ OR psychometry*.ab,ti. OR clinometer*.tw. OR clinometer*.tw. OR Outcome Assessment OR outcome assessment.ab,ti. OR outcome measure*.tw. OR observer variation/ OR observer variation.ab,ti. OR Health Status Indicators/ OR reproducibility of results/ OR reproducible*.ab,ti. OR discriminant analysis/ OR reliab*.ab,ti. OR unreliable*.ab,ti. OR valid*.ab,ti. OR coefficient.ab,ti. OR homogeneity.ab,ti. OR homogeneous.ab,ti. OR internal consistency.ab,ti. OR (cronbach*.ab,ti. AND (alpha.ab,ti. OR alphas.ab,ti.)) OR (item.ab,ti. AND (correlation*.ab,ti. OR selection*.ab,ti. OR reduction*.ab,ti.)) OR agreement.ab,ti. OR precision.ab,ti. OR imprecision.ab,ti. OR precise values.ab,ti. OR test-retest.ab,ti. OR (test.ab,ti. AND retest.ab,ti.) OR (reliab*.ab,ti. AND (test.ab,ti. OR retest.ab,ti.)) OR stability.ab,ti. OR interrater.ab,ti. OR interpreter.ab,ti. OR intrarater.ab,ti. OR intra-rater.ab,ti. OR intertester.ab,ti. OR inter-tester.ab,ti. OR intratester.ab,ti. OR intra-tester.ab,ti. OR interobserver.ab,ti. OR inter-observer.ab,ti. OR intraobserver.ab,ti. OR intraobserver.ab,ti. OR intertechnician.ab,ti. OR inter-technician.ab,ti. OR intratechnician.ab,ti. OR intra-technician.ab,ti. OR interexaminer.ab,ti. OR inter-examiner.ab,ti. OR intraexaminer.ab,ti. OR intraexaminer. ab,ti. OR interassay.ab,ti. OR inter-assay.ab,ti. OR intraassay.ab,ti. OR intra-assay.ab,ti. OR interindividual.ab,ti. OR inter-individual.ab,ti. OR intraindividual.ab,ti. OR intra-individual.ab,ti. OR interparticipant.ab,ti. OR inter-participant.ab,ti. OR intraparticipant.ab,ti. OR intra-participant.ab,ti. OR kappa.ab,ti. OR kappas.ab,ti. OR repeatable*.ab,ti. OR ((replica*.ab,ti. OR repeated.ab,ti.) AND (measure.ab,ti. OR measures.ab,ti. OR findings.ab,ti. OR result.ab,ti. OR results.ab,ti. OR test.ab,ti. OR tests.ab,ti.)) OR generalize*.ab,ti. OR generalist*.ab,ti. OR concordance.ab,ti. OR (intraclass.ab,ti. AND correlation*.ab,ti.) OR discriminative.ab,ti. OR known group.ab,ti. OR factor analysis.ab,ti. OR factor analyses.ab,ti. OR dimension*.ab,ti. OR subscale*.ab,ti. OR (multitrait.ab,ti. AND scaling.ab,ti. AND (analysis.ab,ti. OR analyses.ab,ti.)) OR item discriminant.ab,ti. OR interscale correlation*.ab,ti. OR error.ab,ti. OR errors.ab,ti. OR individual variability.ab,ti. OR (variability.ab,ti. AND (analysis.ab,ti. OR values.ab,ti.)) OR (uncertainty.ab,ti. AND (measurement.ab,ti. OR measuring.ab,ti.)) OR standard error of measurement.ab,ti. OR sensitive*.ab,ti. OR responsive*.ab,ti. OR ((minimal.ab,ti. OR minimally.ab,ti. OR clinical.ab,ti. OR clinically.ab,ti.) AND (important.ab,ti. OR significant.ab,ti. OR detectable.ab,ti.) AND (change.ab,ti. OR difference.ab,ti.)) OR (small*.ab,ti. AND (real.ab,ti. OR detectable.ab,ti.) AND (change.ab,ti. OR difference.ab,ti.)) OR meaningful change.ab,ti. OR ceiling effect.ab,ti. OR floor effect.ab,ti. OR Item response model.ab,ti. OR IRT.ab,ti. OR rausch.ab,ti. OR Differential item functioning.ab,ti. OR DIF.ab,ti. OR computer adaptive testing.ab,ti. OR item bank.ab,ti. OR cross-cultural equivalence.ab,ti.) |

*Online Resource Table 2.*

Criteria for good measurement properties

| Measurement property | Definition | Rating | Criteria | Source |
| --- | --- | --- | --- | --- |
| Reliability | | | | |
| Internal consistency  (CTT methods) | Degree of interrelatedness among items | + | Cronbach alpha(s) > 0.70 | Terwee et al (2007) |
|  |  | ? | Cronbach alpha(s) not determined OR dimensionality unknown OR conflicting evidence |  |
|  |  | - | Cronbach alpha(s) < 0.70 |  |
| Internal consistency  (IRT methods) |  | + | Person Separation Index > 0.70 | Heinl et al (2016) |
|  |  | ? | Person Separation Index not determined |  |
|  |  | - | Person Separation Index <0.70 |  |
| Measurement error |  | + | MIC > SDC  OR MIC outside the LOA | Terwee et al (2007) |
|  |  | ? | MIC not defined |  |
|  |  | - | MIC < SDC  OR MIC equals or inside LOA |  |
| Reliability  (inter-rater and test-retest) | Inter-rater assesses scores from different people at the same time, whilst intra-rater assesses scores from the same person at different times Test-re-test assesses a measures stability over time | + | ICC/weighted Kappa > 0.70 OR Pearson’s r > 0.80 | Heinl et al (2016) |
|  |  | ? | Neither ICC/weighted Kappa or Pearson’s r calculated |  |
|  |  | - | ICC/weighted Kappa > 0.70 OR Pearson’s r > 0.80 |  |
| Validity | | | | |
| Content validity | The degree to which a measure is an adequate reflection of the construct that it intends to measure | + | All items are considered to be relevant for the construct to be measured, for the target population, and for the purpose of the measurement AND the questionnaire is considered to be comprehensive | Terwee et al (2007) |
|  |  | ? | Not enough information available |  |
|  |  | - | Not all items are considered to be relevant for the construct to be measured, for the target population, and for the purpose of the measurement OR the questionnaire is not considered to be comprehensive |  |
| Structural validity  (CTT methods) | Degree to which scores of a measure are an adequate reflection of the dimensionality of the construct to be measured | + | Factors should explain at least 50% of the variance OR CFI or TLI or comparable measure > 0.95 AND (RMSEA < 0.06 OR SRMR < 0.08) | Terwee et al (2007) and modified version available on COSMIN website. |
|  |  | ? | Not all information for ‘+’ reported |  |
|  |  | - | Criteria for ‘+’ not met |  |
| Structural validity  (IRT methods) |  | + | Residual correlations among the items after controlling for the dominant factor < 0.20 OR Q3’s < 0/37, item scalability > 0.30, IRT model fit: G2 > 0.01, no DIF for important subject characteristics (such as age, gender, education): McFadden’s R2 < 0/02, OR no non-uniform DIF | Terwee et al (2007) and modified version available on COSMIN website. |
|  |  | ? | Important statistics not reported |  |
|  |  | - | Criteria for ‘+’ not met |  |
| Hypothesis Testing (convergent/divergent validity) | The degree to which relationships between scores on one measure are sufficiently related (convergent) or unrelated (divergent) to scores on other instruments measuring similar or dissimilar constructs, or different groups of patients (discriminative) | + | Correlations with instruments measuring the same construct > 0.50 OR at least 75% of the results are in accordance with the hypotheses AND correlation with related constructs is higher than with unrelated constructs | Terwee et al (2007) |
|  |  | ? | Solely correlations determined with unrelated constructs |  |
|  |  | - | Correlations with instruments measuring the same construct <0.50 OR < 75% of the results are in accordance with the hypotheses OR correlation with related constructs is higher than with unrelated constructs |  |
| Hypothesis Testing (discriminant/known groups) |  | + | Differences in scores on the measurement instrument for all evaluated patient subgroups are statistically significant OR > 75% of results in accordance with hypotheses | Heinl et al (2016) |
|  |  | ? | Some differences statistically significant, others not |  |
|  |  | - | Differences in scores on the measurement instrument for all evaluated patient subgroups are not statistically significant OR < 75% of results in accordance with hypotheses |  |
| Criterion validity | Degree to which scores of a measure are an adequate reflection of the gold standard | + | Convincing arguments that gold standard is “gold” AND correlation with gold standard > 0.70 OR (sensitivity AND specificity > 70%) | Terwee et al (2007)  de Vet et al (2015) |
|  |  | ? | Not all information for ‘+’ reported |  |
|  |  | - | Criteria for ‘+’ not met |  |
| Responsiveness | The ability to detect change following intervention | + | SDC or SDC < MIC OR MIC outside the LOA OR RR > 1.96 OR AUC > .70 | Modified Terwee et al (2007) |
|  |  | ? | Doubtful design or method |  |
|  |  | - | SDC or SDC > MIC OR MIC equals or inside LOA OR RR < 1.96 OR AUC < .70 despite adequate design and methods |  |

| *Online Resource Table 3*  Quality of measurement properties per questionnaire | | | | | | | | | | |
| --- | --- | --- | --- | --- | --- | --- | --- | --- | --- | --- |
|  | Reliability | | | Hypothesis testing | | | | Responsiveness | | |
| Tool  (number of papers) | Internal Consistency | Test-retest | Inter-rater | Content Validity | Structural Validity | Convergent/ Divergent Validity | Discriminant Validity | Criterion Validity | Stability | Change |
| Parent Reported Child Behaviour | | | | | | | | | | |
| CBCL 1.5-5 year (2) | +++ |  |  |  | --- | - |  |  |  |  |
| CBRS (4) | + |  |  |  | ++ | - |  |  |  |  |
| ECBI (6) | ++ | - |  |  | -- | ++ |  | ++ |  |  |
| IBQ-R (2) | ? |  | ? | ++ | ? |  |  |  |  |  |
| SDQ 2-4 years (2) | +++ |  |  |  | --- |  |  |  |  |  |
| SDQ 3-16 years (3) | +++ |  | -- |  | --- |  |  |  |  |  |
| Parent Reported Social and Emotional Development | | | | | | | | | | |
| BIQ (1) | +++ |  | -- |  | --- | -- |  |  |  |  |
| BITSEA (3) | ++ | ++ | -- |  | ? | ++ |  | -- |  |  |
| PAS-R (1) | +++ |  | -- |  | +++ | - |  |  |  |  |
| Parent Reported Language Development | | | | | | | | | | |
| MCDI i and ii (1) | +++ |  |  |  |  |  |  | +++ |  |  |
| MCDI iii (1) | ++ |  |  |  |  |  | + |  |  |  |
| Practitioner Administered Developmental Test | | | | | | | | | | |
| BSID III (2) |  |  | ? |  |  | + |  | ? |  |  |
| MSEL (1) |  |  |  |  |  | ++ |  |  |  |  |
| NRSLD (1) | ? | - |  |  |  | ? | + |  |  |  |
| NOTE: *Strong level of evidence* (+++ or ---): Consistent findings in multiple studies (2 or more) of good methodological quality or in one study of excellent methodology quality. *Moderate level of evidence* (++ or --): Consistent findings in multiple studies (2 or more) of fair methodological quality or in one study of good methodological quality. *Limited level of evidence* (+ or -): One study of fair methodological quality. *Conflicting level of evidence* (+/-): Conflicting findings. *Unknown* (?): Only studies of poor methodological quality - or criteria not met for + or - in majority of studies  CBCL = Child Behaviour Checklist; CBRS = Child Behaviour Rating Scale; ECBI = Eyberg Child Behaviour Inventory; IBQ-R = Infant Behaviour Questionnaire – Revised; SDQ = Strengths and Difficulties Questionnaire; BIQ = Behavioural Inhibition Questionnaire; BITSEA = Brief Infant Toddler Social and Emotional Assessment; PAS-R = Preschool Anxiety Scale Revised; MCDI = MacArthur Bates Communication Development Inventories; BSID = Bayleys Scales of Infant Development; MSEL = Mullen Scales of Early Learning; NRSLD = New Reynell Developmental Scales of Language. | | | | | | | | | | |
